# A novel short-term high-lactose culture approach combined with a matrix-assisted laser desorption ionization-time of flight mass spectrometry assay for differentiating *Escherichia coli* and *Shigella* species using artificial neural networks

**DOI:** 10.1371/journal.pone.0222636

**Published:** 2019-10-08

**Authors:** Jin Ling, Hong Wang, Gaomin Li, Zhen Feng, Yufei Song, Peng Wang, Hong Shao, Hu Zhou, Gang Chen

**Affiliations:** 1 Department of Biochemical Drugs and Biological Products, Shanghai Institute for Food and Drug Control, Shanghai, China; 2 NMPA Key Laboratory for Quality Control of Therapeutic Monoclonal Antibodies, Shanghai Institute for Food and Drug Control, Shanghai, China; 3 Department of Pharmacy, Zhejiang Jinhua Guangfu Hospital, Jinhua, China; 4 Department of Antibiotics and Microbiology, Shanghai Institute for Food and Drug Control, Shanghai, China; 5 Department of Gastroenterology, Lihuili Hospital of Ningbo Medical Center, Ningbo, China; 6 Shanghai Key Laboratory of Intelligent Information Processing, School of Computer Science, Fudan University, Shanghai, China; 7 Department of Analytical Chemistry, Shanghai Institute of Materia Medica, Chinese Academy of Sciences, Shanghai, China; Fisheries and Oceans Canada, CANADA

## Abstract

**Background:**

*Escherichia coli* is currently unable to be reliably differentiated from *Shigella* species by routine matrix-assisted laser desorption ionization-time of flight mass spectrometry (MALDI-TOF MS) analysis. In the present study, a reliable and rapid identification method was established for *Escherichia coli* and *Shigella* species based on a short-term high-lactose culture using MALDI-TOF MS and artificial neural networks (ANN).

**Materials and methods:**

The *Escherichia coli* and *Shigella* species colonies, treated with (Condition **1**)/without (Condition **2**) a short-term culture with an in-house developed high-lactose fluid medium, were prepared for MALDI-TOF MS assays. The MS spectra were acquired in linear positive mode, with a mass range from 2000 to 12000 Da and were then compared to discover new biomarkers for identification. Finally, MS spectra data sets **1** and **2**, extracted from the two conditions, were used for ANN training to investigate the benefit on bacterial classification produced by the new biomarkers.

**Results:**

Twenty-seven characteristic MS peaks from the *Escherichia coli* and *Shigella* species were summarized. Seven unreported MS peaks, with *m/z* 2330.745, *m/z* 2341.299, *m/z* 2371.581, *m/z* 2401.038, *m/z* 3794.851, *m/z* 3824.839 and *m/z* 3852.548, were discovered in only the spectra from the *E*. *coli* strains after a short-term high-lactose culture and were identified as belonging to acid shock protein. The prediction accuracies of the ANN models, based on data set **1** and **2**, were 97.71±0.16% and 74.39±0.34% (*n* = 5), with an extremely remarkable difference (*p* < 0.001), and the areas under the curve of the receiver operating characteristic curve were 0.72 and 0.99, respectively.

**Conclusions:**

In summary, adding a short-term high-lactose culture approach before the analysis enabled a reliable and easy differentiation of *Escherichia coli* from the *Shigella* species using MALDI-TOF MS and ANN.

## Introduction

Matrix assisted laser desorption-ionization time-of-flight mass spectrometry (MALDI-TOF MS) is a fast and cost-effective method for bacterial identification, and it is used routinely in many laboratories and clinical testing organizations[1–3]. Although standard mass spectrum database-based MALDI-TOF MS can identify thousands of bacterial species, *Escherichia coli* (*E*. *coli*), the most common bacteria in clinical practice, is currently unable to be reliably differentiated from Shigella by routine MALDI-TOF MS analysis [4]. Because the *E*. *coli* and *Shigella* species are closely related and they both belong to the family Enterobacteriaceae, their MS spectra are very similar to each other. The MALDI-TOF MS identification results can be only reported as *E*. *coli*/*Shigella* species, which challenges the entity separation and rapid identification in epidemiology and clinical diseases[5].

*E*. *coli* and *Shigella* species are classified as separate species based on their biochemical characteristics and clinical relevance[6,7]. Assessing the biochemical characteristics and serotyping are commonly used for the identification of *E*. *coli* and *Shigella* species to obtain accurate results. These approaches are classical and reliable, but may have a suboptimal diagnostic performance.

The accurate identification of *E*. *coli* and *Shigella* species isolated from the clinical sample is urgently required for clinical diagnostics and public health. In this study, we present a novel short-term culture approach that is combined a MALDI-TOF MS analysis method to realize the accurate and reliable identification of *E*. *coli* and *Shigella* species.

## Materials and methods

### Bacterial strains

A total of 23 bacterial strains identified by a consensus approach of biochemical and 16S rRNA gene sequencing was selected for the experiment, which covered all the common *Escherichia* and *Shigella* species (See supporting information).

### Culture and sample preparation

**Fig 1:**
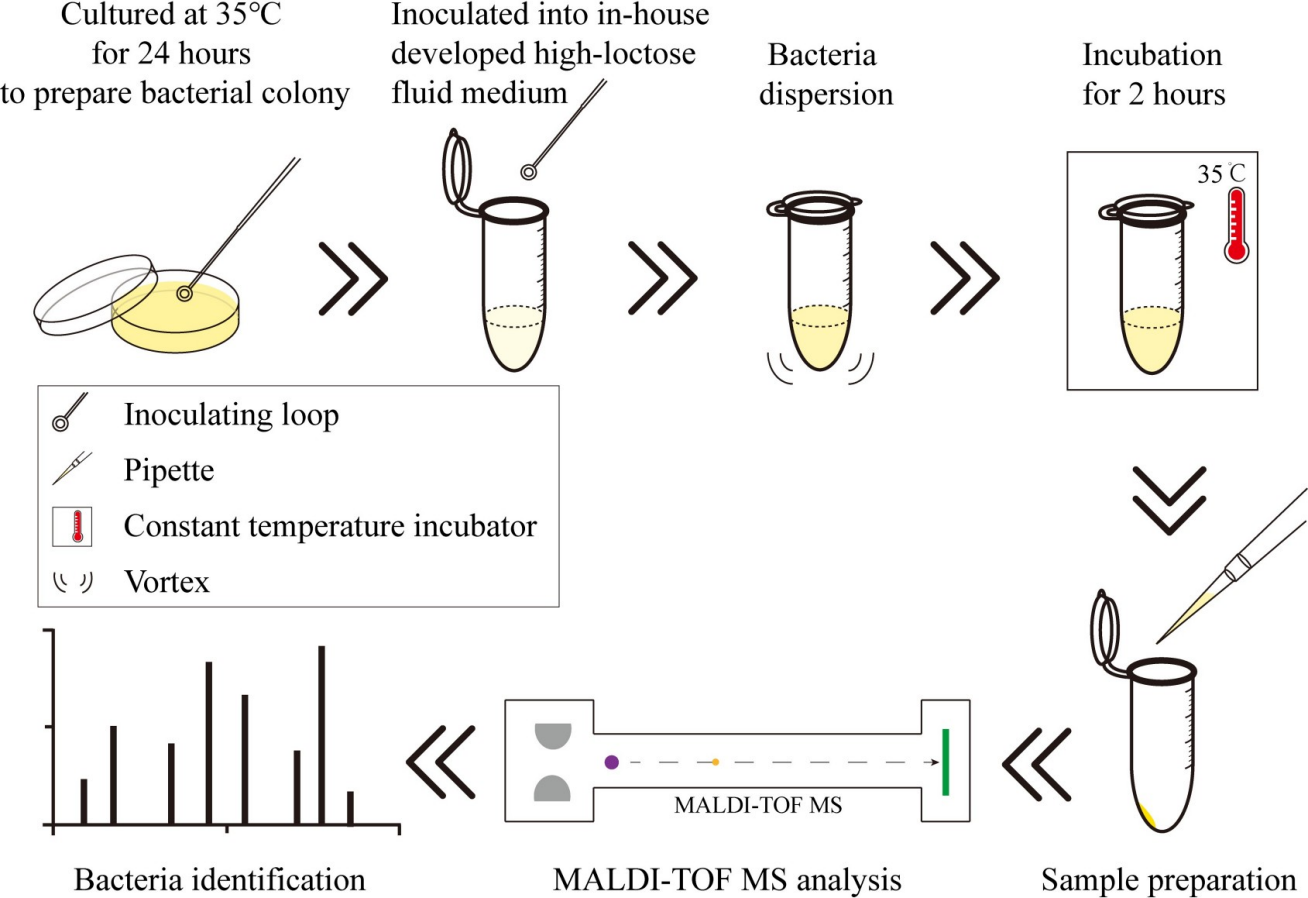
Procedures of the short-term culture approach. The procedures for the short-term culture using the in-house developed high-lactose fluid medium for the differentiation of the *E*. *coli* and *Shigella* species.

The strains were grown on commercial tryptic soy agar (Huankai microbial, Guangzhou, China) at 35°C for 24 h to obtain fresh colonies. The strain colonies were inoculated into our in-house developed high-lactose fluid medium and were incubated at 35°C for 2 h (Fig 1). The colonies on tryptic soy agar and the bacterial suspension in the fluid medium were prepared before the MALDI-TOF MS analysis (See supporting information).

### MALDI-TOF MS data acquisition

The MS analyses and MS/MS analyses were performed using a 4800 Plus MALDI-TOF MS (AB Sciex, Redwood City, US) and an Autoflex maX MALDI-TOF/TOF system (Bruker Daltonik GmbH, Bremen, Germany). The MS spectra were acquired in linear positive-ionization mode. Afterwards, the target MS peak was selected for the MS/MS analysis in a reflector mode. The MS/MS spectra were interpreted primarily with the FlexAnalysis^TM^ software (Bruker Daltonics, Bremen, Germany) (See supporting information).

### Protein identification

The protein database searches were performed with MASCOT version 2.5 (Matrix Science, London, UK) and BioTools 3.2 software (Bruker Daltonics, Bremen, Germany) against the Swiss-Prot database for protein identification (See supporting information).

### Bacterial identification using artificial neural networks

Back propagation neural networks (BPNN), a frequently used artificial neural network, was employed to recognize the classification of the target bacteria, and this was performed in Matlab^TM^ software R2015b (MathWorks, Redick, USA) (See supporting information).

## Results

### Comparison of the spectra in the different culture conditions

**Fig 2:**
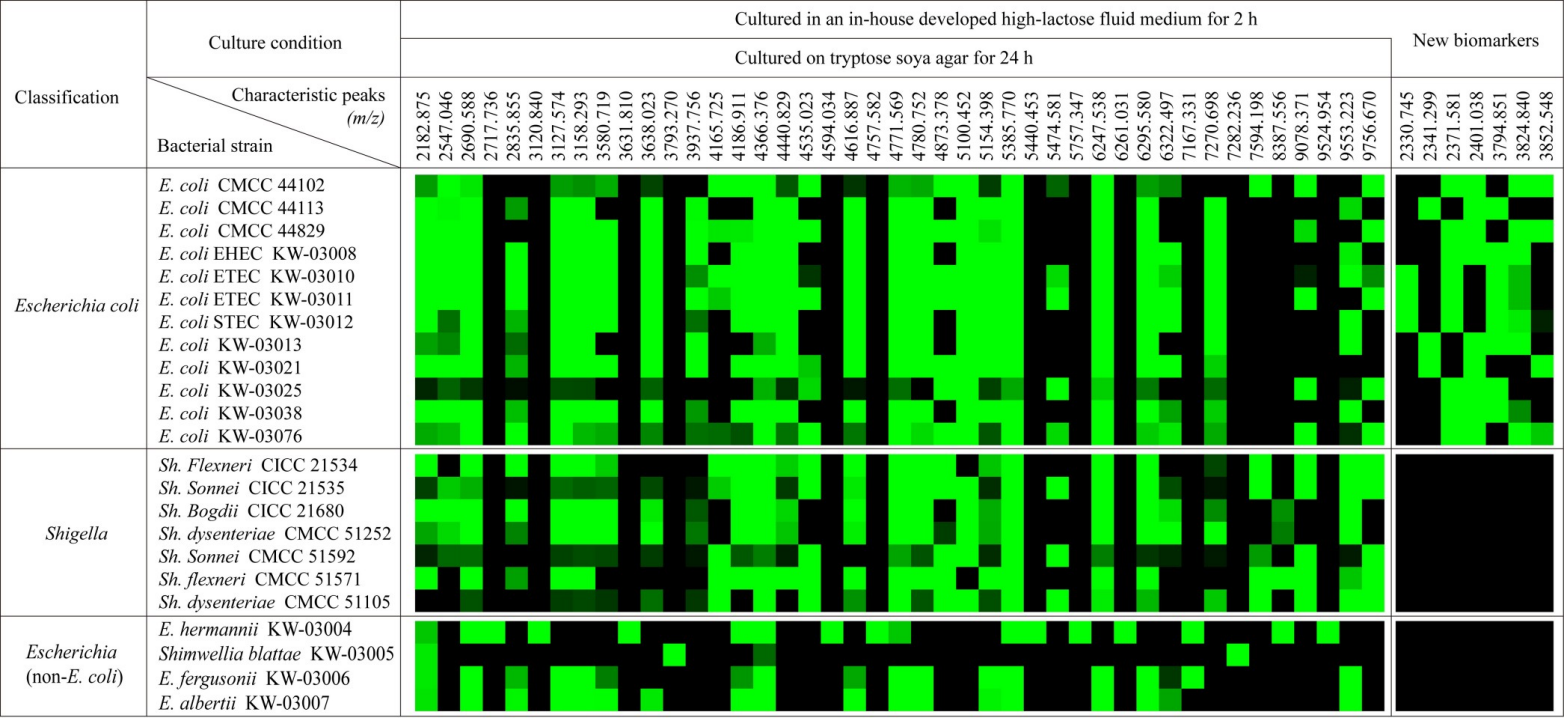
Coloured heat map of the characteristic MS peaks. Coloured heat map showing the characteristic MS peaks in the spectra from the *E*. *coli*, *Shigella* and other four *Escherichia* strains. The relative intensity of the MS peak was categorized using Hierarchical Clustering Explorer 3.0. Blocks, with a relative intensity equal to 0 shown in black; blocks with a relative intensity less than 10% are shown in dark green; blocks with a relative intensity between 10% to 100% are shown in bright green. *E*., *Escherichia*; *Sh*., *Shigella*.

As shown in Fig 2, the spectra from *E*. *coli* strains obtained from the tryptic soy agar culture condition contained 27 fully conserved MS peaks, which were also shared with most of the *E*. *coli* strains. Analogously, the 27 MS peaks described above were also observed in the spectra of most of the *Shigella* strains. There was no significant differential peak between the spectra from the *E*. *coli* strains and the *Shigella* strains, leading an inability to identify them using MALDI-TOF MS (S1 Fig). In addition, compared with the spectra from the *E*. *coli* and *Shigella* species, the spectra from other 4 *Escherichia* species did not contain MS peaks of *m/z* 2547, *m/z* 3937, *m/z* 4165, *m/z* 4186, *m/z* 4440, *m/z* 4535, *m/z* 4873, *m/z* 5100, *m/z* 5474, *m/z* 7594, *m/z* 9078 and *m/z* 9756 simultaneously (Fig 2).The spectrum from *E*. *hermannii* showed special MS peaks of *m/z* 2717, *m/z* 3120, *m/z* 3631, *m/z* 4594, *m/z* 4757, *m/z* 5440, *m/z* 5757, *m/z* 6261 and *m/z* 9524, which were not observed in the spectra of any of the other strains and could be used as biomarkers for identification. The MS peaks of *m/z* 3793 and *m/z* 7282 were only observed in the spectrum from *Shimwellia blattae*, which could be used as biomarkers for identification. Based on the characteristic peaks described above, the other *Escherichia* species was easily identified using MALDI-TOF MS.

The spectra from the *E*. *coli* strains changed after culture in the in-house developed high-lactose fluid medium for 2 hours. In addition to the 27 fully conserved MS peaks, seven new peaks of *m/z* 2330.745, *m/z* 2341.299, *m/z* 2371.581, *m/z* 2401.038, *m/z* 3794.851, *m/z* 3824.839 and *m/z* 3852.548 were obviously observed in the spectra from the *E*. *coli* strains (S2 Fig). In contrast, these differential MS peaks were not observed in either the *Shigella* or the other *Escherichia* species, as shown in S2 Fig. Therefore, these seven newly discovered characteristic MS peaks could be used as novel biomarkers to differentiate the *E*. *coli* from *Shigella* species.

### Identification of new characteristic MS peaks

As shown in Table 1, six of the seven new characteristic MS peaks were successfully identified both by the amino acid sequence and by the type of protein. By searching the protein databases, these amino acid sequences belonged to acid shock protein, which indicated that the new characteristic peaks were fragments of acid shock protein. Because of the acid shock protein mutation, the molecular weights of the matched protein were different, and the new characteristic MS peaks had similar mass-to-charge ratios.

**Table 1:** Identification of the new biomarkers.

Precursor ion (*m/z*)	Fragment ion (*m/z*)	Amino acid sequence	Mascot score	Protein hits	Molecular weight	Source
**2330.288**	110.023, 297.132, 481.225, 618.253, 792.443, 1016.466, 1153.556, 1281.567, 1409.626, 1538.686, 1705.813, 1794.886, 2073.129, 2241.410, 2330.293	AAKKHAGKHSHQQPAKPAAQPAA	160	Acid shock protein	10470	Escherichia coli (strain SE11)
**2341.174**	129.095, 266.173, 403.227, 518.331, 645.380, 801.433, 986.471, 1114.576, 1227.525, 1314.648, 1442.673, 1540.671, 1641.820, 1805.906, 1942.970, 2070.947, 2195.317	AAKKHHKNAKAEQKAPEQKAQ	144	Acid shock protein	10525	Escherichia coli O6:K15:H31 (strain 536 / UPEC)
**2371.207**	129.066, 266.125, 355.106, 518.263, 645.311, 801.376, 999.411, 1144.520, 1227.462, 1344.574, 1472.574, 1570.542, 1671.747, 1835.845, 1972.790, 2225.229	AAKKHHKNTKAEQKAPEQKAQ	139	Acid shock protein	10585	Escherichia coli (strain K12 / MC4100 / BW2952)
**2401.201**	129.035, 266.007, 368.102, 518.225, 625.263, 801.417, 982.469, 1144.500, 1224.586, 1352.602, 1480.758, 1672.278, 1776.871, 1865.676, 2003.033, 2144.293, 2367.983,	AAKKHAKKHSHQQPAKPAAQPAA	114	Acid shock protein	10585	Escherichia coli (strain K12 / MC4100 / BW2952)
**3794.908**	265.917, 394.219, 636.342, 1049.585, 1433.869, 1570.899, 1707.990, 1910.008, 2485.891, 2205.954, 2741.652, 3216.909	AETATTPAPTATTTKAAPAKTTHHKKQHKAAPAQKAQU	173	Acid shock protein	10555	Escherichia coli O17:K52:H18 (strain UMN026 / ExPEC)
**3824.807**	266.038, 394.245, 522.368, 929.552, 1049.620, 1433.860, 1570.967, 1708.022, 1910.160, 2206.128, 2348.200, 2516.133, 2908.195, 3035.718	AETTTTPAPTATTTKAAPAKTTHHKKQHKAAPAQKAQK	101	Acid shock protein	10585	Escherichia coli (strain K12 / MC4100 / BW2952)
**3852.973**	154.393, 266.032, 522.326, 929.495, 1305.738, 1433.849, 1570.936, 1707.997, 1910.457, 2038.033, 2206.097, 2347.966, 2415.776, 2671.798, 2909.116, 3065.072, 3418.344, 3674.481	No matched result	-	No matched result	-	-

### Adding a short-term high-lactose culture approach benefits back propagation neural networks classification modelling

**Fig 3:**
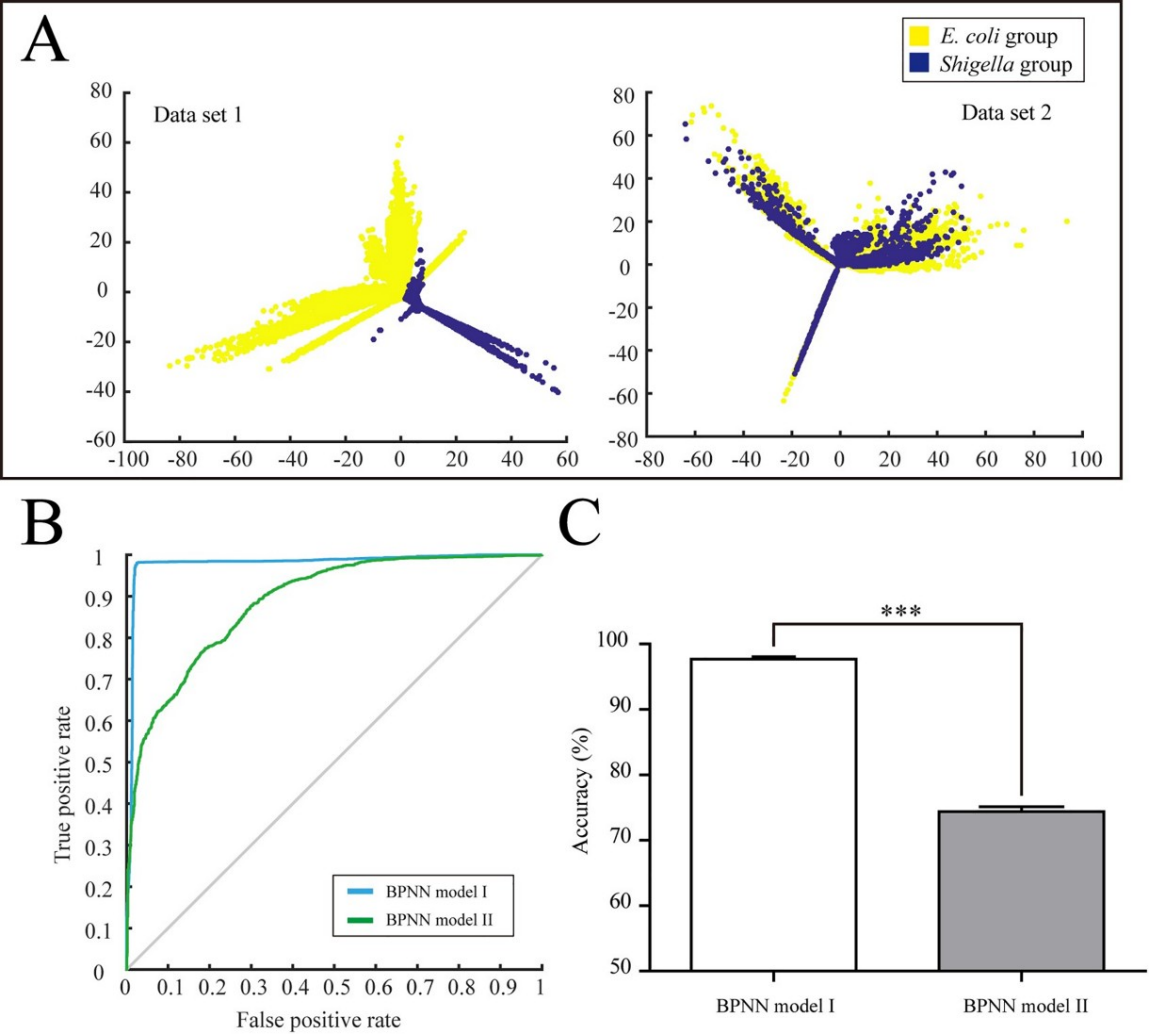
Results of the bacterial identification using artificial neural networks. (A) Scatter-plot of data set **1** and **2** after dimensionality reduction with the isomap algorithm. (B) Receiver operating characteristic curves of the BPNN model Ⅰ and Ⅱ were plotted based on the matching extent between the predicted value and the label value. (C) The accuracy was calculated by model simulation using random test samples. The values are the mean ± SD; ***, *p* < 0.001 compared with the BPNN model Ⅱ group, *n* = 5 independent runs. BPNN, back propagation neural network.

Data sets **1** and **2**, with the same matrix size of 12350×12396, were processed with the isomap algorithm to reduce their dimensionality from 12396 to 2048. As shown in Fig 3A, after the dimensionality reduction, Data set **1** obviously separated into two clusters, with *E*. *coli* group and *Shigella* group labels. Whereas Data set **2** was not clustered with satisfaction. The results indicated that Data set **1** contained prominent characteristics of dimensionality for distinguishing spectrum from the *E*. *coli* and *Shigella* species. BPNN Model I and II were trained basing Data set **1** and **2**, with identical parameters. The overview of the receiver operating characteristic curves for the two BPNN models, achieved by the synchronously optimizing BPNN model I and II, are shown in Fig 3B. The areas under curve value were 0.99 and 0.72 for the BPNN model I and II, respectively. The classification accuracies of BPNN model I and II were 97.71±0.16% and 74.39±0.34% (n = 5). There was an extremely remarkable difference between two accuracy results, which were compared with a *t*-test (*p* < 0.001) (Fig 3C). These results suggested that BPNN model I was significantly improved when a short-term high-lactose culture approach was added before the MS analysis.

## Discussion

Most *E*. *coli* strains are part of the normal gut flora, whereas the *Shigella* species are considered to be pathogenic bacteria. The *Shigella* species has been separated from *E*. *coli* strains, as a requirement for clinical diagnostics, using biochemical methods and serological techniques, since the first *Shigella* species were discovered in 1898[8]. A series of distinct phenotypic and biochemical characteristics-based laboratory methods are commonly used to distinguish them, which are currently not efficient enough to satisfy the diagnostic requirements. By comparing genomes and housekeeping genes, the *E*. *coli* and *Shigella* species are be considered to be part of the same phylogenetic continuum rather than clearly distinct species. The nuances, at the molecular level, between the *E*. *coli* and *Shigella* species lead to an indistinguishableness by routine sequencing of the 16S rRNA gene and MALDI-TOF MS-based identification[8].

The MALDI-TOF MS technique is a highly cost-effective and time-efficient way to identify bacteria[9–11]. Due to their close relatedness, the spectra of *E*. *coli* and *Shigella* species have a high degree of similarity. In our investigation, the *E*. *coli* and *Shigella* species shared 27 main characteristic MS peaks and had no significantly differentiated MS peaks. A routine MALDI-TOF MS analysis for the identification of *E*. *coli* and *Shigella* species is difficult. A recent study showed that a specialized automated system, based on an analysis of selective biomarker MS peaks in the spectra, using Biotyper software followed by an analysis with FlexAnalysis and ClinProTools software for the identification of *E*. *coli* and *Shigella* species, was better than traditional techniques, including an automated microbiology identification system and serotyping[12]. Paauw et al. created a high-resolution reference library for implementation in Biotyper software in order to reflect the genetic diversity of the *E*. *coli* and *Shigella* species and to rapidly distinguish the *Shigella* species from *E*. *coli*[13]. However, the reliability of the identification largely depended on the quality and resolution of the spectra, especially the sample spectra. In routine MALDI-TOF MS analysis, the quality of the sample spectra is usually affected by a series of factors, including the type of sample, bacterial state, culture medium recipe, sample pretreatment method and operation and the parameters of the MS analysis[14]. Even if a high-quality spectrum is acquired, the detection and recognition of low intensity biomarkers for the identification of the *E*. *coli* and *Shigella* species is still difficult. To provide a reliable and efficient identification method for the *E*. *coli* and *Shigella* species for clinical diagnostic purposes, the biochemical method and the MALDI-TOF MS technique are combined. A novel high-lactose fluid medium is prepared on the optimized prescription. We added a short-term culture approach, using an in-house developed high-lactose fluid medium, before the routine MALDI-TOF MS assay, which enabled a reliable distinction between the *E*. *coli* and *Shigella* species. Seven MS peaks were newly observed in the spectra of the *E*. *coli* species only, which can be used as new biomarkers to distinguish *E*. *coli* and *Shigella* using MALDI-TOF MS.

The limited strains involved in our study and the unknown biomarkers may cause methodological limitations of its application. The distinct mechanism needs to be understood to determine whether the present approach successfully applies to any of the *E*. *coli* and *Shigella* strains. The proposed biomarkers are identified as fragments of acid shock protein, which makes the distinct mechanism clarified. Acid shock protein is encoded by the *asr* gene in *E*. *coli*, which is strongly induced by a high acid environment (pH < 5.0)[15]. Acid shock protein is subject to an N-terminal cleavage of the signal peptide, yielding an 8-kDa polypeptide, which is detected in acid-shocked bacteria. These results indicated that the new biomarkers emerged because of *E*. *coli* was under an acid shock condition. To our knowledge, *E*. *coli* strains can ferment lactose, by which organic acids are produced [16]. These organic acids are dissolved in the liquid medium, leading a pH reduction of the culture condition, and thus, the *E*. *coli* species are stressed to produce many acid shock proteins to protect themselves against the low pH survival condition made by the lactose fermentation [15]. After 2 h incubation, the pH value of liquid medium in *E*.*coli* group was 5.2±0.5, which support our hypothesis. Whereas the *S*. *flexneri*, *S*. *sonnei*, *S*. *bogdii E*. *hermannii*, *Shimvellia blattae*, *E*. *fergusonii* and *E*. *albertii* strains do not ferment lactose, while some *S*. *dysenteriae* strains ferment lactose slowly (delayed lactose fermentation), which leads to no significant change of pH, as well as the expression of acid shock protein when they survive in a high-lactose condition for a short time. Therefore, our method can be widely applied to differentiate all lactose-fermenting *E*. *coli* from *Shigella* species and other non-lactose-fermenting *Escherichia* species.

The classification algorithm plays a critical role in the MALDI-TOF MS-based bacterial identification method[17–19]. BPNN, as an artificial intelligence algorithm, has to be well-trained using high-quality data with a good separation[20,21]. This gives a satisfying classification result and achieves automatic bacterial identification. Although the new biomarkers are obvious and easily recognized by an analyst, the BPNN model test on the new biomarkers is required. The aim of the BPNN model establishment is to determine whether the new biomarkers provide a high feature separation of the data and further improve the classification efficiency and accuracy. BPNN model I and II were synchronously optimized to investigate the influence of adding a short-term high-lactose approach. The results indicated that the feature separation of data set **1** was higher than that of data set **2**, and BPNN model I performed better than BPNN model II. The *E*. *coli* and *Shigella* species were correctly classified, with 97.71±0.16% accuracy, using the BPNN model I. This encourages us to recommend this method for the identification of *E*. *coli* and *Shigella* species in the clinical laboratory. The short-term culture, combined with the MALDI-TOF MS assay, described here provides a novel bacteria identification strategy for allied bacteria groups, which are unable to be identified using the routine MALDI-TOF MS approach.

## Conclusion

In the present study, we provide a novel short-term culture approach, with optimized high-lactose fluid medium components, before the MS analysis in order to induce the expression of new biomarkers to distinguish *E*. *coli* and *Shigella* using a MALDI-TOF MS. The new biomarkers increase the data separation, which significantly improves the classification efficiency and accuracy of artificial neural networks.

## Supporting information

S1 Fig The MALDI-TOF MS spectra of the *E*. *coli*, *Shigella* and other *Escherichia* strains cultured on tryptic soy agar for 24 h. The MALDI-TOF MS spectra of the *E*. *coli*, *Shigella* and other *Escherichia* strains acquired from the linear positive mode, with a mass range from 2000 to 12000 Da. All the experimental strains were cultured on tryptic soy agar for 24 h followed by sample preparation and MALDI-TOF MS analysis.
(TIF)

S2 Fig The MALDI-TOF MS spectra of the *E*. *coli*, *Shigella* and other *Escherichia* strains treated with an additional 2 h culture in the in-house developed high-lactose fluid medium. The MALDI-TOF MS spectra of the *E*. *coli*, *Shigella* and other *Escherichia* strains acquired from the linear positive mode, with mass range from 2000 to 12000 Da. All the experimental strains were cultured on tryptic soy agar for 24 h, with an additional 2 h culture in the in-house developed high-lactose fluid medium followed by sample preparation and MALDI-TOF MS analysis. Newly discovered MS peaks, as identification biomarkers, are marked with the respective mass-to-charge ratio.
(TIF)

S1 File Supporting information. **The detailed methods of experiments in research work.** The bacterial strains for experiments, culture conditions, sample preparation method, MALDI-TOF MS data acquisition method, protein identification method and artificial neural networks for bacterial identification were detailed.
(DOCX)
