# Supplementary figures and images for: A novel short-term high-lactose culture approach combined with a matrix-assisted laser desorption ionization-time of flight mass spectrometry assay for differentiating Escherichia coli and Shigella species using artificial neural networks
Source: PLoS One. 2019 Oct 8;14(10):e0222636. doi: 10.1371/journal.pone.0222636 (PMC6782097; doi:10.1371/journal.pone.0222636)

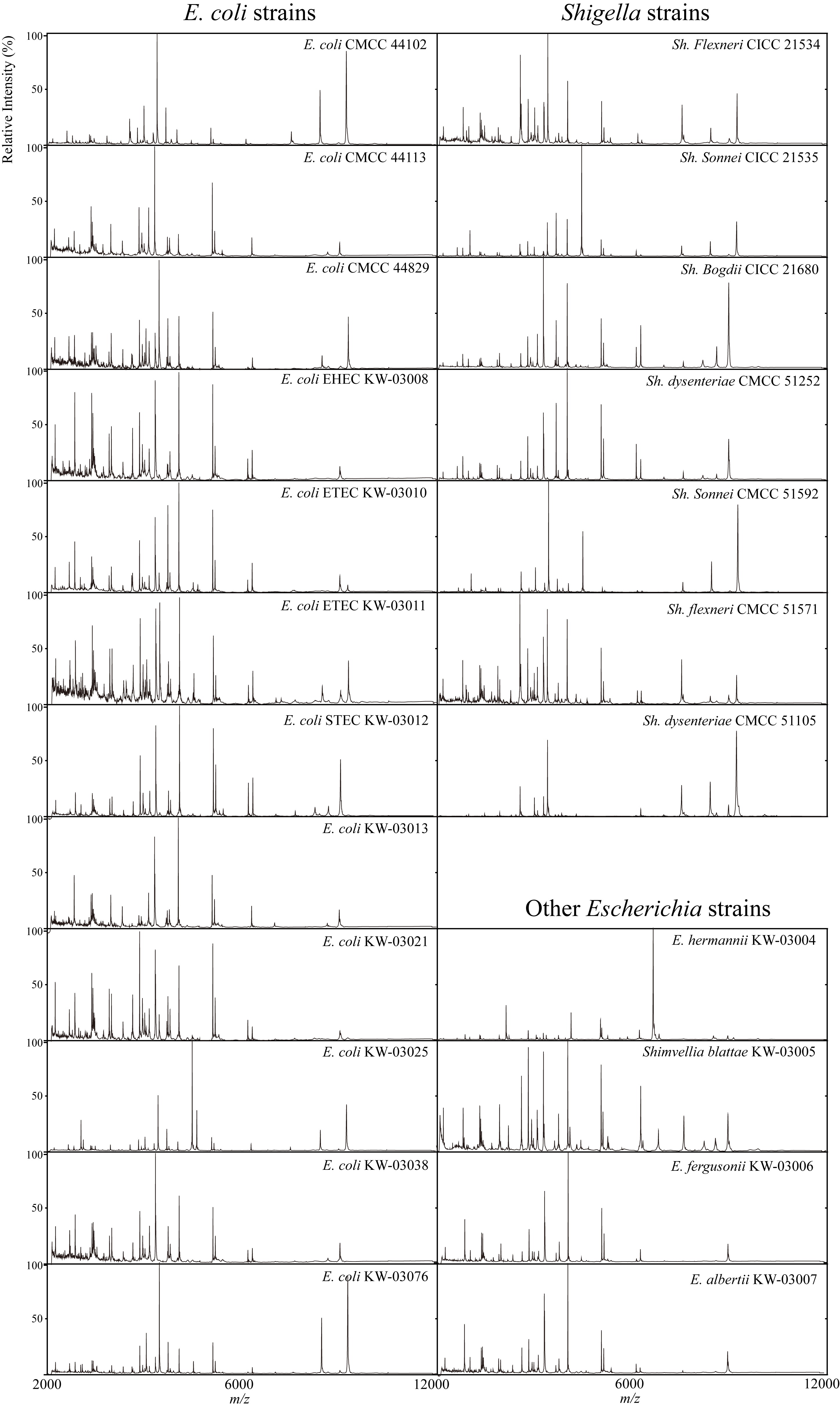

Supplement: S1 Fig — The MALDI-TOF MS spectra of the E. coli, Shigella and other Escherichia strains acquired from the linear positive mode, with a mass range from 2000 to 12000 Da. All the experimental strains were cultured on tryptic soy agar for 24 h followed by sample preparation and MALDI-TOF MS analysis. (TIF) [file pone.0222636.s001.tif]

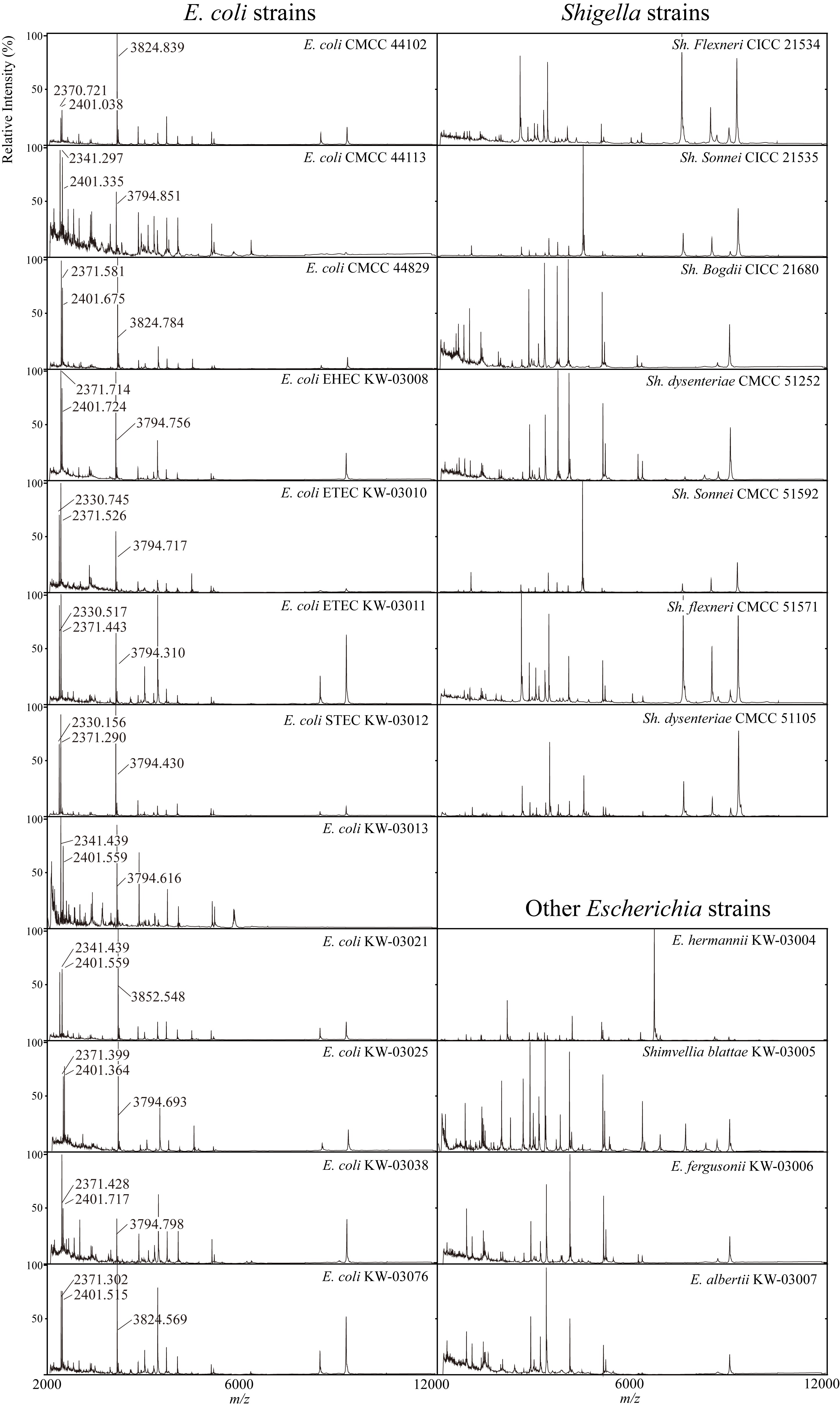

Supplement: S2 Fig — The MALDI-TOF MS spectra of the E. coli, Shigella and other Escherichia strains acquired from the linear positive mode, with mass range from 2000 to 12000 Da. All the experimental strains were cultured on tryptic soy agar for 24 h, with an additional 2 h culture in the in-house developed high-lactose fluid medium followed by sample preparation and MALDI-TOF MS analysis. Newly discovered MS peaks, as identification biomarkers, are marked with the respective mass-to-charge ratio. (TIF) [file pone.0222636.s002.tif]
