# Supplementary material for: A novel short-term high-lactose culture approach combined with a matrix-assisted laser desorption ionization-time of flight mass spectrometry assay for differentiating Escherichia coli and Shigella species using artificial neural networks
Source: PLoS One. 2019 Oct 8;14(10):e0222636. doi: 10.1371/journal.pone.0222636 (PMC6782097; doi:10.1371/journal.pone.0222636)
Supplement: S1 File — The detailed methods of experiments in research work. The bacterial strains for experiments, culture conditions, sample preparation method, MALDI-TOF MS data acquisition method, protein identification method and artificial neural networks for bacterial identification were detailed. (DOCX) [file pone.0222636.s003.docx]

**A novel short-term high-lactose culture approach combined with a matrix-assisted laser desorption ionization-time of flight mass spectrometry assay for differentiating *Escherichia coli* and *Shigella* species using artificial neural networks**

# Supporting information

## Bacterial strains

A total of 23 bacterial strains, including 7 *Shigella* strains (2 *S. dysenteriae*, 2 *S. flexneri*, 2 *S. sonnei* and 1 *S. bogdii*) and 12 *E. coli* strains (7 *E. coli*, 1 *E. coli STEC*, 2 *E coli EHEC* and 2 *E. coli ETEC*) and 4 other *Escherichia* strains (1 *E. hermanaii*, 1 *Shimvellia blattae*, 1 *E. fergusouii*, 1 *E. albertii*), were selected for the experiment. Nine of the 12 *E. coli* strains and 4 other *Escherichia* strains isolated from food samples in our laboratory were identified as *E. coli* (KW-03013, KW-03021, KW-03025 and KW-03076), *E. coli* STEC (KW-03012), *E. coli* EHEC (KW-03008 and KW-03038), *E. coli* ETEC (KW-03010 and KW-03011), *E. hermanaii* (KW-03004), *Shimvellia blattae* (KW-03005), *E. fergusouii* (KW-03006) and *E. albertii* (KW-03007). Three of the 12 *E. coli* strains and all 7 *Shigella* strains were CMCC and CICC reference strains of *E. coli* (CMCC 44102, CMCC 44113 and CMCC44829), *S. dysenteriae* (CMCC 51252 and CMCC 51105), *S. flexneri* (CMCC 51571 and CMCC 21534), *S. sonnei* (CMCC 51592 and CICC 21535) and *S. bogdii* (CICC 21680) (Table S1).

**Table S1 Classification and source of experimental bacterial strains**

| Genus | Species | Strains | Source |
| --- | --- | --- | --- |
| *Escherichia* | *E. coli* | *E. coli* KW-03013 | Isolated strain |
|  |  | *E. coli* KW-03021 | Isolated strain |
|  |  | *E. coli* KW-03025 | Isolated strain |
|  |  | *E. coli* KW-03076 | Isolated strain |
|  |  | *E. coli* CMCC 44102 | Reference strain |
|  |  | *E. coli* CMCC 44113 | Reference strain |
|  |  | *E. coli* CMCC 44829 | Reference strain |
|  |  | *E. coli* STEC KW-03012 | Isolated strain |
|  |  | *E. coli* EHEC KW-03008 | Isolated strain |
|  |  | *E. coli* EHEC KW-03038 | Isolated strain |
|  |  | *E. coli* ETEC KW-03010 | Isolated strain |
|  |  | *E. coli* ETEC KW-03011 | Isolated strain |
|  | *E. hermanaii* | *E. hermanaii* KW-03004 | Isolated strain |
|  | *E. fergusouii* | *E. fergusouii* KW-03006 | Isolated strain |
|  | *E. albertii* | *E. albertii* KW-03007 | Isolated strain |
|  | *Shimvellia blattae (E. blattae)* | *Shimvellia blattae* KW-03005 | Isolated strain |
| *Shigella* | *S. dysenteriae* | *S. dysenteriae* CMCC 51252 | Reference strain |
|  |  | *S. dysenteriae* CMCC 51105) | Reference strain |
|  | *S. flexneri* | *S. flexneri* CMCC 51571 | Reference strain |
|  |  | *S. flexneri* CMCC 21534 | Reference strain |
|  | *S. sonnei* | *S. sonnei* CMCC 51592 | Reference strain |
|  |  | *S. sonnei* CICC 21535) | Reference strain |
|  | *S. bogdii* | *S. bogdii* CICC 21680 | Reference strain |

## Culture and sample preparation

The strains were inoculated onto commercial tryptic soy agar (Huankai microbial, Guangzhou, China) followed by a 24 h incubation at 35℃ to obtain the bacterial standard spectrums. After the 24 h incubation, the fresh colonies were extracted with 60 μL of 70% formic acid (Sigma-Aldrich, Louis, USA) and an equal volume of acetonitrile (Merck, Darmstadt, Germany). After centrifugation at 10000 g for 3 min, 1 μL of the lysate was loaded onto a MALDI target plate spot and left to dry. Each sample spot was overlaid with 1 μL α-Cyano-4-hydroxycinnamic acid (CHCA) (Sigma-Aldrich, Louis, USA) in 50% acetonitrile/2% trifluoroacetic acid (Tedia, Fairfield, USA) matrix and was dried at room temperature. For the short-term high-lactose culture, the strains were inoculated into 100 μL of the in-house developed high-lactose fluid medium, containing MacConkey fluid medium (Huankai microbial, Guangzhou, China) and a 10% aseptic α-lactose (Sinopharm Chemical Reagent Company, Shanghai, China) solution (3:7, *v*:*v*), followed by an incubation at 35℃ for 2 h (Fig. 1). After the 2 h incubation, the bacterial suspension was centrifuged at 8000 g for 3 min, and the supernatant was carefully removed by aspiration. The bacterial precipitation was prepared in the same manner as that of fresh colonies, which was described above before the MALDI-TOF MS analysis.

## MALDI-TOF MS data acquisition

The spectrum was obtained by summing 50 acceptable sub-spectrums obtained in random sampling mode, with a fixed laser intensity of 3500 for the MS analysis on a 4800 Plus MALDI-TOF MS (AB Sciex, Redwood City, US). The raw data were collected between 2000 and 12000 *m/z* in the linear positive-ionization mode. The peak detection parameters were set as follows: Signal/Noise > 20; local noise windows width = 250 and minimum peak width at full width half max = 2.9.

For the MS/MS analyses, the sample analyses were first operated in reflector mode for the MALDI-TOF MS spectra acquirement on an Autoflex maX MALDI-TOF/TOF system (Bruker Daltonik GmbH, Bremen, Germany). Then, the target MS peaks with *m/z* of 2330.288, 2341.174, 2371.207, 2401.201, 3794.908, 3824.807 and 3852.973 were selected for the MS/MS analysis in sequence. The data were accumulated from 1000 consecutive laser shots. A mixture of nine peptides, ranging from *m/z* 757.400 to 3147.470, was used for the sample calibration.

## Protein identification

The search parameters were set as follows: mass tolerance of ±100 ppm for the parent ion and ±0.7 Da for the fragment ion, and the species was restricted to bacteria. The protein identification was inferred from matching the identified amino acid sequence. The ion score was -10×Log(*P*), where *P* is the probability that the observed match is a random event. The individual ion scores > 59 indicate identity or extensive homology (*p*<0.05).

## Bacterial identification using artificial neural networks

Six hundred and fifty spectra of each strain collected from treating with/without the in-house developed high-lactose culture, grouped as **1** and **2,** were involved in the back propagation neural networks (BPNN) modelling. Data set **1** and **2** were extracted intact from the spectra of the target bacteria in group **1** and **2**, respectively. The data sets of the full spectra were reduced dimensionally using the isomap nonlinear dimensionality reduction algorithm. The input number was set as 2048 for the BPNN model training. After parameter optimization, the corresponding numbers of neurons of the hidden layer were finally set as 320 for the first hidden layer and 120 for the second hidden layer. The transfer functions of the hidden layer and output layer and the network training function were defined as hyperbolic tangent sigmoid transfer function, linear transfer function and Levenberg-Marquardt function, respectively. At each modelling process, 70% of the spectra were selected randomly for the model training, half of the rest of the spectra were selected randomly for cross-validation, and the other 15% of the spectra were used to test the model. The training data were performed with 300 epochs, at maximum, for the establishment of the BPNN model. A cross-validation approach was used to reduce the risk of “over fit,” and the model accuracy results were calculated based on 5 rounds of testing.
